# Patient and Doctor Delays in Smear-Negative and Smear-Positive Pulmonary Tuberculosis Patients Attending a Referral Hospital in Istanbul, Turkey

**DOI:** 10.1155/2014/158186

**Published:** 2014-10-14

**Authors:** Gulbanu Horzum Ekinci, Esra Karakaya, Esra Akkutuk Ongel, Osman Haciomeroglu, Adnan Yilmaz

**Affiliations:** Department of Pulmonology, Sureyyapasa Center for Chest Diseases and Thoracic Surgery Training and Investigation Hospital, 34854 Istanbul, Turkey

## Abstract

*Objectives.* To measure delays from onset of symptoms to initiation of treatment in patients with smear-negative and smear-positive pulmonary tuberculosis and to identify reasons for these delays.* Methods.* A total of 136 newly diagnosed pulmonary tuberculosis patients were interviewed using a structured questionnaire.* Results.* The patients were divided into two groups. Group 1 included 65 smear-negative patients. There were 71 smear-positive patients in group 2. The median application interval was 10 days in group 1 and 14 days in group 2. While 24.6% of the patients had patient delay in group 1, patient delay was present in 33.8% of the patients in group 2 (*P* > 0.05). The median health care system interval was 41 days in group 1 and 16 days in group 2 (*P* < 0.0001). The most common reason for patient delay was neglect of symptoms by patient in both groups. A low index of suspicion for tuberculosis by physicians was the most common reason for doctor delays.* Conclusions.* Delays are common problem in smear-negative and smear-positive pulmonary tuberculosis patients. Delays should be reduced to reach an effective tuberculosis control. Education of public and physicians about tuberculosis is the most important effort to reduce delays.

## 1. Introduction

At the beginning of the twenty-first century, tuberculosis remains an important public health problem worldwide. It was estimated that almost one-third of the global population was infected by the tuberculosis bacillus in 2006 [1]. The latest estimates of The World Health Organization included that there were 8.6 million newly diagnosed tuberculosis cases and 1.3 million deaths from this disease in 2012. One of the main targets in tuberculosis control programs is to reduce tuberculosis transmission in the community. Early diagnosis of the disease and prompt initiation of treatment are two key factors to reach an effective tuberculosis control [2, 3]. Delays in diagnosis and treatment result in more extensive disease, more complications, higher costs, and increased mortality. They also lead to an increased period of infectivity in the community [2, 4]. It has been estimated that undetecting and untreating a single infectious person can infect more than ten people every year [5]. Delays in the diagnosis and treatment of tuberculosis patients have been commonly reported in both high and low prevalence countries [2–7]. In Turkey, the incidence, prevalence, and mortality rates were of 28, 24, and 3.1 per 100 000 in 2010, respectively [8]. Our hospital, located in Istanbul, is a referral hospital for chest diseases and tuberculosis. This center provides to tuberculosis patients who have been referred district health care facilities. The aims of present study were to measure delays from onset of symptoms to initiation of treatment in patients with smear-negative and smear-positive pulmonary tuberculosis and to identify reasons for these delays.

## 2. Materials and Methods

This cross-sectional study was conducted at Sureyyapasa Center for Chest Diseases and Thoracic Surgery Training and Investigation Hospital, consisting of 605 beds. It is one of Turkey's main referral hospitals for tuberculosis, receiving patients from the various health centers in the district as well as patients from all over the country. We investigated all the patients with tuberculosis diagnosed in two tuberculosis clinics between January 2010 and October 2010. The patients with extrapulmonary tuberculosis, those who had previous histories of tuberculosis treatment, the patients who had no symptoms, pulmonary tuberculosis patients below 15 years of age, the patients who did not accept responding the questionnaire, and those who did not remember some dates in their disease course were excluded from the study. The standard procedures recommended by the National Tuberculosis Control Program in the diagnosis of pulmonary tuberculosis were used. Smear-positive pulmonary tuberculosis is confirmed when there are at least two acid-fast-bacilli- (AFB-) positive smear results or when one sputum specimen is positive for AFB in addition to radiographic abnormalities consistent with active pulmonary tuberculosis. Smear-negative pulmonary tuberculosis is diagnosed in a patient with three initial negative smear examinations by direct microscopy for AFB and who has failed to respond to a course of broad spectrum antibiotics and again three smear-negative examinations by direct microscopy and X-ray abnormalities suggestive of active tuberculosis as determined by a treating physician. Smear-negative tuberculosis can also be diagnosed in a patient with three initial smear examinations negative by direct microscopy but positive by culture. The present study included 136 pulmonary tuberculosis patients who had the inclusion criteria. The 136 patients included in the study were divided into two groups. Group 1 consisted of 65 smear-negative pulmonary tuberculosis patients. Group 2 included 71 patients with smear-positive pulmonary tuberculosis. Consent was obtained from each patient fully explaining the purpose and nature of the study.

Clinical files of the patients were analyzed by 2 authors of this study, and a questionnaire was created to obtain data by the interview with the patients. For each patient, the following information was gathered based on these data: sex, age, educational level, smoking habit, presence of index case for tuberculosis, presence of comorbidity, symptoms, the onset time of initial symptoms, time of first doctor visit, time of admission to our hospital, and time of treatment initiation. The following time intervals and delays were determined for each patient:* the patient's application interval* was defined as the time interval between the onset of symptoms and the first doctor visit. Intervals that exceeded 30 days were considered indicative of a* patient delay* in both groups.* The referral interval* was defined as the time from the first doctor visit to admission. With regard to our health care system, intervals that exceeded two days were considered indicative of a* referral delay* in both groups [2].* Clinical interval* was defined as the time from admission to initiation of treatment. While intervals that exceeded 13 days were considered indicative of a* clinic delay *in group 1, a period of more than two days was indicative of a* clinical delay* in group 2.* Health care system interval* (referral interval + clinical interval) was defined as the time from the first doctor visit to initiation of treatment. While intervals that exceeded 15 days were considered indicative of a* doctor delay* in group 1, a period of more than four days was indicative of a* doctor delay* in group 2. The* total interval* was the time between the onset of symptoms and time of treatment initiation. While intervals that exceeded 45 days were considered indicative of a* total delay* in group 1, a period of more than 34 days was indicative of a* total delay* in group 2 [2, 9]. The reasons for the delays were also evaluated.

### 2.1. Statistical Analysis

The chi-square test, ANOVA test, or Student's *t*-test were used to assess differences between groups.

## 3. Results

During the study period, we evaluated 136 patients with pulmonary tuberculosis. The 136 patients were divided into two groups. Group 1 consisted of 65 smear-negative pulmonary tuberculosis patients. There were 27 females and 38 males in group 1. The mean age of the patients was 36.9 years (range 16–68 years). Group 2 included 71 patients with smear-positive pulmonary tuberculosis. There were 29 females and 42 males in this group. The mean age was 35.8 years (range 16–67 years).  Table 1 summarizes demographic characteristics of the groups. There were no significant differences between group 1 and group 2 (*P* > 0.05).

Table 2 presents the values of the intervals. The median application interval was 10 days in group 1 and 14 days in group 2 (*P* > 0.05). Smear-negative patients had longer health care system interval than smear-positive patients (*P* < 0.01). The median referral interval was 13 days in group 1 and 8 days in group 2. There was no significant difference between the two groups (*P* > 0.05). Clinical interval was longer in group 1 than in group 2 (*P* = 0.0022). Smear-negative patients had longer total interval than smear-positive patients (*P* = 0.0318).

The rate of patient delay was 24.6% and 33.8% in group 1 and group 2, respectively (*P* > 0.05). Only 12 (18.5%) smear-negative patients have started treatment within 15 days of the first doctor visit. According to delay criteria, there was a doctor delay in 81.5% of the patients in this group. Health care system interval was shorter than 4 days in 16 smear-positive patients. In group 2, 55 (77.5%) patients had a doctor delay. There was no significant difference between group 1 and group 2 according to the rate of doctor delay. The number of the patients having clinical delay was higher in group 1 than in group 2 (*P* = 0.0394). The rate of total delay was 66.1% and 62% in group 1 and group 2, respectively (*P* > 0.05).  Table 3 shows distribution of the patients having delay.

Table 4 summarizes reasons for patient and doctor delays. Sixteen smear-negative patients had a patient delay. We identified 18 reasons for patient delay in 16 patients. We found 30 reasons for patient delay in 24 smear-positive patients having a patient delay. The most common reason for patient delay was neglect of symptoms by the patient in two groups. There were 74 reasons for doctor delay in 53 smear-negative patients with a doctor delay. We identified 59 reasons for doctor delay in 55 smear-positive patients with a doctor delay. A low index of suspicion for pulmonary tuberculosis by the physician and underutilized or delayed sputum examinations for acid-fast smears were the most common reasons for doctor delay.

## 4. Discussion

Although tuberculosis is both preventable and curable, it remains a leading infectious cause of morbidity and mortality worldwide [5, 9]. The primary goal of tuberculosis control programs is to minimize transmission within the community and reduce tuberculosis incidence by detecting and treating active tuberculosis disease as early as possible [3–5]. Tuberculosis infection is predominantly spread by patients with pulmonary tuberculosis. Although it is widely believed that smear-negative cases are less infectious than smear-positive cases, it was reported that at least 17% of tuberculosis cases were the result of transmission from smear-negative tuberculosis cases [10]. Delays in the diagnosis and treatment of pulmonary tuberculosis result in a prolonged period of infectivity in the community [3, 5]. It is well known that delays are common problem in both developed and developing countries [11].

Delays in the diagnosis and treatment of smear-positive patients have been commonly investigated by authors in previous studies. Nevertheless, the number of studies evaluating delays in smear-negative patients is limited [3, 11]. Investigators have described several delays from onset of symptoms to treatment. Delays are divided into patient, doctor or health care system, and total delays. Referral delay, diagnosis delay, treatment delay, and clinical delay are subgroups of doctor delay [2, 6, 12]. The present study included both smear-negative and smear-positive pulmonary tuberculosis patients. We evaluated patient delay, doctor delay, referral delay, clinical delay, and total delay in our study. While intervals that exceeded 30 days were considered indicative of a patient delay in the majority of previous studies [2, 7, 9], there are no universally accepted criteria for doctor delay and its subgroups [3, 6]. For smear-positive patient group, we used delay criteria described by authors in a previous study from Turkey [2]. While we used the same criteria for patient delay and referral delay in smear-negative patient group, we measured clinical delay, doctor delay, and total delay by using different cut-off points [2, 9].

We found that smear-negative and smear-positive patients had similar application intervals and the rates of patient delay. The median application interval for smear-negative and smear-positive patients was 10 and 14 days, respectively. Sherman et al. [13] reported that the median patient delay was 27 days in smear-negative patients and 21 days in smear-positive patients. In a Norway study, the median application interval was 14 and 28 days in these patients, respectively [6]. For smear-positive patients, shorter (0.3 weeks) or extremely longer (120 days) median application intervals were reported in previous studies [14, 15]. A previous study from Turkey noted that the median application interval was found to be 17.5 days in smear-positive patients with pulmonary tuberculosis and 34.8% of these patients had patient delay [2]. It is known that the length of application interval may be associated with several factors. Characteristics of the patients such as sex [16], age [13, 17], symptoms [2], education level [15], the patients' area of residence [17], and economic status [2] may affect the length of patient delay. We conclude that the patients' characteristics may be reason for different application intervals among studies.

Smear-negative and smear-positive patients had a similar referral interval values in our study whereas we found that clinical interval, health care system interval, and total interval were longer in smear-negative patients than in smear-positive patients. For smear-negative patients, the median referral, clinical, health care system, and total intervals were 13, 19, 41, and 61 days, respectively. For smear-positive patients, these intervals were 8, 2, 16, and 48 days, respectively. Lorent et al. [3] reported that smear-negative pulmonary tuberculosis was associated with a longer health care system delay. The median health care system delay was 31 days for smear-negative patients and 6 days for smear-positive group in a previous report [13]. Whitehorn et al. [12] reported that patients with smear-negative disease were 2.81 times more likely to have experienced total delay than those with smear-positive disease. For smear-negative patients, the rates of delay were 73.8% for referral delay, 60% for clinical delay, and 81.5% for doctor delay in the present study. For smear-positive patients, these rates were 68.4%, 40.8%, and 77.5%, respectively. A previous report suggested that the rates of delay were 81.9% for referral delay and 87.8% for doctor delay in smear-positive patients [2]. While smear-negative and smear-positive patients had similar the rates of referral delay and doctor delay in our study, the rate of clinical delay was higher in smear-negative patients. Our results indicated that the length of clinical interval was more significant than the length of referral interval in smear-negative patients. It is known that smear-negative pulmonary tuberculosis is difficult to diagnose and often requires assessing the response to antibiotic therapy as well as reviewing radiological and other investigations. These may result in longer clinical and health care system intervals [3, 12]. We conclude that longer health care system and total intervals resulted from long clinical interval in smear-negative patients.

Several possible reasons for patient delay and doctor delay were described in previous reports. Characteristics of the patients such as sex, age, level of education, economic status, smear status, distance to a physician or health center, patients' area of residence, presence of index case for tuberculosis and symptoms may affect the length of patient and doctor delays [2, 13, 15–17]. Okur et al. [18] noted that common possible reasons for patient delay were neglect of symptoms by the patient, sociocultural factors, distance to health center, and economic status. In our series, the most common reason for patient delay was neglect of symptoms by the patients in both group 1 and group 2. A low index of suspicion for tuberculosis and underutilized or delayed sputum smear examinations for acid-fast bacilli were the most common reasons for doctor delay in our series. It was reported that underutilized chest X-ray examinations, underutilized or delayed sputum smear examinations, a low degree of suspicion for tuberculosis, and health care system and laboratory system delays were the most common reasons for doctor delays [2, 18]. A previous report showed that the general practitioners' low level of suspicion of tuberculosis and their failure to take expeditious action in either ordering proper investigations or referring the patient to government hospitals contributed in a major way to doctor's delay. The patients often needed to return to the same doctors or to seek the advice of other doctors because of the persistence of their symptoms. Thus, the number of doctors consulted and the number of doctor's consultations before referral to hospital are high [19]. Underutilization of chest X-ray examination is a common problem among physicians. Doctor delay is shortened when patients are examined by chest X-ray [20]. Tuberculosis patients are commonly given antibiotics prior to tuberculosis treatment [3, 12, 21]. It was reported that patients who received antibiotics prior to tuberculosis diagnosis had a diagnostic delay that was twice as long as that of patients who did not receive antibiotics [21]. An assessment of the response to antibiotic therapy is common approach in the diagnosis of smear-negative patients and it may result in a clinical and health care system delay [21, 22].

The present study has some limitations. First, the study was conducted in a referral hospital and only patients who presented themselves or those who were referred were included; therefore it would be illogical to generalize the findings to the whole country. Second, there may have been some recall bias from patients. Third, for measuring the length of patient's application interval and referral interval, we depended on the reply of the patient. Because the patients provided retrospective responses, the durations of these intervals may have been underestimated or overestimated in some patients.

In conclusion we identified several delays from onset of symptoms to treatment in pulmonary tuberculosis patients. Smear-negative and smear-positive tuberculosis patients have a similar rate of patient delay. Doctor delay is more significant in smear-negative tuberculosis patients than in smear-positive tuberculosis patients. Prolonged delays increase the risk of infection transmission in the community. Therefore, delays should be reduced through several efforts to reach an effective tuberculosis control. Since a low index of suspicion for tuberculosis among physicians and neglect of symptoms by the patients are common problem, physicians and public should be educated about tuberculosis. Improvement of laboratory system delays and health care system delays is the other important effort to reduce delays.

## Figures and Tables

**Table 1:** Demographic characteristics of the study population.

	Group 1		Group 2	
	*n*	%	*n*	%
Sex^~^				
Male	38	58.5	42	59.2
Female	27	41.5	29	40.8
Mean age (range)	36.9 (16–68) years		35.8 (16–67) years	
Education level^†^				
Primary school	29	44.6	42	59.2
High school	26	40.0	23	32.4
University	10	15.4	6	8.4
Smoking habit∗				
Nonsmoker	27	41.5	23	32.4
Ex-smoker	13	20.0	15	21.1
Smoker	25	38.5	33	46.5
History of index case for tuberculosis∗∗				
Yes	30	46.2	31	43.7
No	35	53.8	40	56.3
Comorbidity°				
Yes	15	23.1	22	31.0
No	50	76.9	49	69.0
Presenting symptom^‡^				
Cough	38	58.5	59	83.1
Sputum production	26	40.0	41	57.7
Fever	17	26.2	22	31.0
Hemoptysis	7	10.8	22	31.0
Chest pain	17	26.2	15	21.1
Dyspnea	8	12.3	4	5.6
Weight loss	14	21.5	29	40.8
Sweating	16	24.2	21	29.6
Other	29	44.6	32	45.1

^~^ *P* > 0.05; ^†^ *P* > 0.05; ∗*P* > 0.05; ∗∗*P* > 0.05; °*P* > 0.05; and ^‡^ *P* > 0.05.

**Table 2:** The values associated with several intervals (days).

Interval	*n*	Mean	SD	Median	95% CI
Application interval^†^					
Group 1	65	29.4	49.4	10	17.2–41.7
Group 2	71	31.7	40.8	14	22.1–41.4
Health system interval∗					
Group 1	65	64.5	85.8	41	43.2–85.8
Group 2	71	33.0	47.1	16	21.8–34.1
Referral interval∗∗					
Group 1	65	26.5	34.9	13	17.9–35.2
Group 2	71	25.2	41.5	8	15.3–35.0
Clinical interval°					
Group 1	65	38.2	79.1	19	18.5–57.8
Group 2	71	7.5	23.3	2	2–13
Total interval^‡^					
Group 1	65	99.8	116.1	61	71–128.6
Group 2	71	64.7	60.8	48	50.3–79.1

^†^ *P* > 0.05; ∗*P* < 0.01; ∗∗*P* > 0.05; °*P* = 0.0022; and ^‡^ *P* = 0.0318.

**Table 3:** Distribution of the patients having delay with respect to delay criteria in groups.

Presence of delay	Group 1		Group 2	
	*n*	%	*N*	%
Patient delay^†^				
Yes	16	24.6	24	33.8
No	49	75.4	47	66.2
Doctor delay∗				
Yes	53	81.5	55	77.5
No	12	18.5	16	22.5
Referral delay∗∗				
Yes	48	73.8	45	63.4
No	17	26.2	26	36.6
Clinical delay°				
Yes	39	60	29	40.8
No	26	40	42	59.2
Total delay^‡^				
Yes	43	66.1	44	62
No	22	33.9	27	38

^†^ *P* > 0.05; ∗*P* > 0.05; ∗∗*P* > 0.05; °*P* = 0.0394; and ^‡^ *P* > 0.05.

**Table 4:** Possible reasons for patient and doctor delay.

Reason	Group 1		Group 2	
	*n*	%	*n*	%
Patient delay				
Neglect of symptoms by the patient	10	55.6	19	63.3
Distance to health center	2	11.1	2	6.7
Sociocultural factors	2	11.1	6	20
Economic status	2	11.1	2	6.7
Other	2	11.1	1	3.3
Total	**18**	**100**	**30**	**100**
Doctor delay				
Underutilized or delayed sputum examinations	31	41.9	15	25.4
A low index of suspicion for tuberculosis	27	36.5	18	30.5
Reasons associated with patient	8	10.8	7	11.8
Reasons associated with laboratory system	5	6.8	8	13.6
Reasons associated with health care system	3	4	8	13.6
Other	—	—	3	5.1
Total	**74**	**100**	**59**	**100**

## References

[1] Sudre P., Dam G. T., Kochi A. (1992). Tuberculosis: a global overview of the situation today. *Bulletin of the World Health Organization*.

[2] Güneylioglu D., Yilmaz A., Bilgin S., Bayram Ü., Akkaya E. (2004). Factors affecting delays in diagnosis and treatment of pulmonary tuberculosis in a tertiary care hospital in Istanbul, Turkey. *Medical Science Monitor*.

[3] Lorent N., Mugwaneza P., Mugabekazi J., Gasana M., Van Bastelaere S., Clerinx J., Van Den Ende J. (2008). Risk factors for delay in the diagnosis and treatment of tuberculosis at a referral hospital in Rwanda. *International Journal of Tuberculosis and Lung Disease*.

[4] Sendagire I., van der Loeff M. S., Mubiru M., Konde-Lule J., Cobelens F. (2010). Long delays and missed opportunities in diagnosing smear-positive pulmonary tuberculosis in Kampala, Uganda: a cross-sectional study. *PLoS ONE*.

[5] Ayuo P. O., Diero L. O., Owino-Ong’or W. D., Mwangi A. W. (2008). Causes of delay in diagnosis of pulmonary tuberculosis in patients attending a referral hospital in Western Kenya. *East African Medical Journal*.

[6] Farah M. G., Rygh J. H., Steen T. W., Selmer R., Heldal E., Bjune G. (2006). Patient and health care system delays in the start of tuberculosis treatment in Norway. *BMC Infectious Diseases*.

[7] Leutscher P., Madsen G., Erlandsen M., Veirum J., Ladefoged K., Thomsen V., Wejse C., Hilberg O. (2012). Demographic and clinical characteristics in relation to patient and health system delays in a tuberculosis low-incidence country. *Scandinavian Journal of Infectious Diseases*.

[8] World Health Organization (2011). Global tuberculosis control. *WHO Report*.

[9] Lusignani L. S., Quaglio G., Atzori A., Nsuka J., Grainger R., da Conceiçao Palma M., Putoto G., Manenti F. (2013). Factors associated with patient and health care system delay in diagnosis for tuberculosis in the province of Luanda, Angola. *BMC Infectious Diseases*.

[10] Behr M. A., Warren S. A., Salamon H., Hopewell P. C., Ponce De Leon A., Daley C. L., Small P. M. (1999). Transmission of *Mycobacterium tuberculosis* from patients smear-negative for acid-fast bacilli. *The Lancet*.

[11] Storla D. G., Yimer S., Bjune G. A. (2008). A systematic review of delay in the diagnosis and treatment of tuberculosis. *BMC Public Health*.

[12] Whitehorn J., Ayles H., Godfrey-Faussett P. (2010). Extra-pulmonary and smear-negative forms of tuberculosis are associated with treatment delay and hospitalisation. *International Journal of Tuberculosis and Lung Disease*.

[13] Sherman L. F., Fujiwara P. I., Cook S. V., Bazerman L. B., Frieden T. R. (1999). Patient and health care system delays in the diagnosis and treatment of tuberculosis. *International Journal of Tuberculosis and Lung Disease*.

[14] Lienhardt C., Rowley J., Manneh K., Lahai G., Needham D., Milligan P., McAdam K. P. W. J. (2001). Factors affecting time delay to treatment in a tuberculosis control programme in a sub-Saharan African country: the experience of The Gambia. *International Journal of Tuberculosis and Lung Disease*.

[15] Wandwalo E. R., Mørkve O. (2000). Delay in tuberculosis case-finding and treatment in Mwanza, Tanzania. *International Journal of Tuberculosis and Lung Disease*.

[16] Mfinanga S. G., Mutayoba B. K., Kahwa A., Kimaro G., Mtandu R., Ngadaya E., Egwaga S., Kitua A. Y. (2008). The magnitude and factors associated with delays in management of smear positive tuberculosis in Dar es Salaam, Tanzania. *BMC Health Services Research*.

[17] Yimer S., Bjune G., Alene G. (2005). Diagnostic and treatment delay among pulmonary tuberculosis patients in Ethiopia: a cross sectional study. *BMC Infectious Diseases*.

[18] Okur E., Yilmaz A., Saygi A., Selvi A., Süngün F., Öztürk E., Dabak G. (2006). Patterns of delays in diagnosis amongst patients with smear-positive pulmonary tuberculosis at a teaching hospital in Turkey. *Clinical Microbiology and Infection*.

[19] Liam C. K., Tang B. G. (1997). Delay in the diagnosis and treatment of pulmonary tuberculosis in patients attending a university teaching hospital. *International Journal of Tuberculosis and Lung Disease*.

[20] Aoki M., Mori T., Shimao T. (1992). Studies on factors influencing patient’s, doctor’s and total delay of tuberculosis case-finding process of Tuberculosis in Korea. *The International Journal of Tuberculosis and Lung Disease (IJTLD)*.

[21] Golub J. E., Bur S., Cronin W. A., Gange S., Sterling T. R., Oden B., Baruch N., Comstock G. W., Chaisson R. E. (2005). Impact of empiric antibiotics and chest radiograph on delays in the diagnosis of tuberculosis. *International Journal of Tuberculosis and Lung Disease*.

[22] Tattevin P., Che D., Fraisse P., Gatey C., Guichard C., Antoine D., Paty M. C., Bouvet E. (2012). Factors associated with patient and health care system delay in the diagnosis of tuberculosis in France. *International Journal of Tuberculosis and Lung Disease*.

